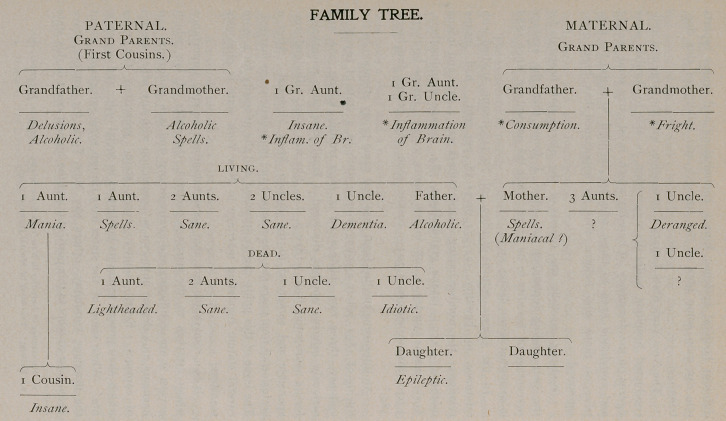# Degeneracy1President’s address at the thirty-first annual meeting of the Medical Association of Central New York, at Auburn, October 18, 1898.

**Published:** 1898-11

**Authors:** William C. Krauss

**Affiliations:** Buffalo, N. Y.


### BUFFALO MEDICAL JOURNAL.

VOL. XXXVIII. NOVEMBER, 1898. NO. 4

### Original Communications.

### DEGENERACY.[1]

BY WILLIAM C. KRAUSS, M. D., Buffalo, N. Y.

MEETING in a city with a fixed population of 1,200, whose term of residence is fixed by law and many of whom being degenerates, it has seemed appropriate to discuss briefly with you today the meaning of degeneracy, its stigmata, and to suggest some remedies for its mitigation. In treating of this subject I am but following out the line of thought suggested in the admirable addresses of my two predecessors in office, which reflects the increasing activity displayed by physicians, and supplemented by the endeavors of reformers and legislators.

The Association of Assistant Physicians of Hospitals for the Insane has recently taken up this subject and from the rich store of material at command should accomplish excellent results from the standpoint of the psychical degenerate; criminologists, especially of the Italian and German schools, have certainly made wonderful progress in this direction. Medical literature teems with essays on this subject, while jurists, legislative bodies and charity organisation societies the world over are considering plans and devising laws which shall have for an object the amelioration and protection of the degenerate, thus relieving and protecting society from its most dangerous and deadly evil.

Morel's definition of the degenerate, expressed over fifty years ago, is as follows: He said that a degenerate is one whose brain and nervous system are unstable from inherited or acquired taint in the parents, who has in consequence undergone imperfectly the embryological changes to a higher type in tissues or organs, and

1. President's address at the thirty-first annual meeting of the Medical Association of Central New York, at Auburn, October 18, 1898.

therefore exhibits tendencies liable to extinguish the race as a type under the usual conditions of the struggle for existence.

Morel was interested chiefly in the study of the most pronounced types of degeneracy, as the idiot, the cretin, the imbecile, and the like, while the new school composed of such men as Lombroso, Benedikt, Ellis, Marro, Krafft-Ebing and others extended the scope and range of their investigations so as to include the criminal, the lunatic, the sexual pervert and other classes of lesser degrees of degeneration.

Morel speaks of the existence of a primitive perfect type of the human race and calls it the master-work and sum of the creation. Degeneration is for him a pathological deviation from this biblical primitive type, a degradation of the progeny. While the physiological deviations are due to the influence of the climate, the nourishment and the habits of life, the pathological deviation or degeneration is due to exaggerations and abnormalities of these influences.

The originally perfect man has been swept away by the doctrine of evolution, and in his stead the new school has set up as the perfect man he who is free as possible from the characteristic features of phylogenetically less mature types. Everything that reminded strongly of possible ancestors of a lower degree was stamped as atavistic and these features are prominently present in those classes of defective or abnormal development.

Many observers, seeing that the pendulum is swinging too far toward atavism, are approaching the problem, as Adolf Meyer[1] so clearly writes, from a point of view similar to this: The so-called normal type is an arbitrary assumption and embraces a large number of physiological variations. It remains to be seen whether certain variations of form or function by themselves or in groups constitute actual signs of degeneration, *i. e.*, whether they are signs of constitutional inferiority with a tendency to become more marked in the offspring.

The degenerate must be considered solely and alone upon the physical, mental and moral stigmata which brand him as an abnormal or atypical man, and prevent him from exerting himself to the highest limit commensurate with his skill and development.

To classify the various stigmata of the degenerate, we may divide the life-history of the individual into three epochs:

1. Americal Journal of Insanity, January, 1896.

(1) *The Pre-natal Epoch.*—Embracing teratological evidence of degeneracy.

(2) *Post-natal Epoch.*—Where the evidence is purely subjective or physical and functional.

(3) *The Post-developmental Epoch.*—Where the evidence is mainly objective or pyschical.

I.—*Pre-natal Epoch.*

The writer is at present unprepared to state positively in how far teratology is to be considered as evidence of degeneracy, but would go on record as stating that the causes underlying degeneration from a physical and psychical standpoint are in the majority of cases identical with those upon which the science of teratology rests.

II.—*Post-natal Epoch.*

Physical stigmata. Objective evidence.

We may divide the physical stigmata into two subdivisions:

(*a*) Morphological deviations from the normal.

1. Deviations of the general proportions of the body—the hands and arms, the feet and the legs, the trunk, the neck, the head as a whole and its various parts, skull, face, nose, mouth, jaws, and the like, may be too small or too large in proportion to the rest of the body, or the stature of the body itself may be out of proportion to the age of the individual.

2. Peculiar forms of special parts—as skull, face, ears, palate, jaws, lips, nose, eyes, brain, thorax and extremities.

(*b*) Functional deviations from the normal.

1. Lack of functional activity of the general organs of the body.

2. Lack of functional activity of the special organs.

3. Developmental irregularities, including habits.

III.—*Post-developmental Epoch.*

Psychical stigmata. Subjective evidence.

The psychical evidences of degeneration may be divided into the (1) Mental stigmata. (2) Moral stigmata. (3) Sensual stigmata.

To take up the various stigmata—physical and psychical—in the short time at my disposal would be utterly impossible and in passing I can only refer to the work done on this subject by several

American authors[1] which harmonise to a remarkable degree. Knowing the character of the various stigmata and their significance the question may well be asked, what constitutes a degenerate and who are the unfortunate individuals or classes ?

The old saying, one swallow does not make a Summer, might be paraphrased into, one symptom does not make a degenerate, but the existence of a combination of degenerate symptoms must be given proper attention.

All the systems of the body and not one system only must be studied and investigated before our judgment reaches a decision. Perhaps not one individual in a hundred can be found who does not offer one symptom of degeneracy, one sign of atavism. Some proportion or percentage must be arbitrarily taken, therefore, to decide who is and who is not a degenerate. Dana says about 2 per cent. of normal men have the stigmata of degeneration, while among lunatics, criminals, abortive types of paranoia and primary forms of neurasthenia the percentage is about 30.

Talbot says " an individual is not a degenerate who possesses only one deformity, but those persons who have three or four may be considered as such."

Berry writes that single defects in certain portions may have no direct signification, but the coëxistence of several, involving to a greater or less degree important parts, is indicative of and associated with other physical or moral deteriorations. As Benedikt truly affirms, they are to be viewed as an anthropological variety of their species, at least among the cultured races. Some writers take five as the criterion and draw the line between the two, and this is perhaps the best rule to observe.

The degenerate classes, therefore, consist of idiots, cretins, imbeciles, lunatics, especially the paranoiac maniac and dement, mutes, epileptics, choreics—chronic and congenital, athetotics—chronic and congenital, hysterics, neurasthenics, chronic criminals, prostitutes, sexual perverts, moral and spiritual perverts.

Turning now to the remedial consideration of the subject, we are at the very outset confronted with obstacles and barriers well-nigh insurmountable. The question of " personal liberty," guaranteed by the constitution of the United States, is thought to be

1. Talbot, E. S., Chicago, Ill.
Peterson, Frederick : State Hospitals Bulletin, July, 1896.
Krauss, William C. : American Journal of Insanity, July, 1898

involved in some of the means proposed for the abatement of this evil. But " personal liberty " was never meant to imply " personal license," and the satisfaction of one's desires and caprice contrary to the dictates of social and moral customs, acting detrimentally and working injury to the community or commonwealth, is not tolerable in the present-day civilisation. It is not my purpose to pose as a reformer, but simply as an agitator, for although the world clamors for reform, yet it hates and ridicules a reformer.

Two great factors make up the sum of human life—heredity and environment. By these the characters of individuals or of generations are molded. The perversion of these two factors constitute the two chief causes of degeneracy and their antidotes are (1) prevention and (2) redemption.

*Prevention.*—The methods proposed bear a close relationship to the causes operating to produce degenerates, and first and foremost of these is heredity.

Heredity has been defined as that peculiar property of an organism which transmits to its offspring the characteristics of its progenitors. If those characteristics are ones of grace, beauty and strength, the offspring will inherit the corresponding qualities of the parent; if, on the other hand, defect and infirmity are the characteristics of the sire, then these qualities will reappear in the young, often with renewed impetus and reinforcement. Moreover, while it is an easy matter for the higher and nobler attributes to become through custom and environment deflected and deteriorated, it is almost an impossibility for the baser and decrepit qualities and conditions to become regenerated and rehabilitated.

These laws hold good not only in the human family but in the vegetable and animal worlds as well; they follow exactly the same course and terminate at the same place. Out of propagated weakness there cannot come strength; out of defects there cannot come perfection. To me heredity is nothing more than a mirror reflecting from one generation to another the grace, beauty and strength, or else the course, ugly, defective features of the one standing before it.

The prevention of conception, therefore, among the degenerate classes and the exercise of control by the commonwealth over the marriages of the unfit, are the two means proposed to thwart the iron grasp of heredity.

The increased complexity of our civilisation indicates the survival, not of the fittest, but of the *unfittest*, and these in their turn—with the seeds of insanity and criminality lurking in their organisation—marry and propagate disease and madness. Marriage, in short, is not physiologically considered, and only too often the most mercenary and sordid considerations are factors in uniting two people altogether unsuited to one another, and whose marriage is the consummation of physical ruin. On the other hand, owing to selfishness and self-indulgence, marriage in some sections of the community is long deferred, and the unnatural suppression of a natural instinct is a prime cause of deterioration, mental worry, and very frequently moral degeneration.

The existence of purely criminal families in which crime has been a distinguishing feature through many generations has been frequently commented on. Thomson cites an example in which there were six convicts in a single household. Déspine quotes a case of three brothers ; the first had seven children, of which there were five thieves and one murderer ; the second had two children, both of whom were murderers ; while the only child of the third was both a thief and a murderer.[1]

The history of the Juke family, as unearthed by Dugdale,[2] gives some idea of the terrible influence of heredity. He calculated that the descendants of one individual, making a family of 1,200 strong, entailed upon the community during a period of seventy-five years an amount of loss and expense equal to $1,250,000. The history of many of these individuals could be traced, and of these 180 were paupers and vagabonds and seventy-six criminals, while of the women, as far as the sixth generation, 52.40 per cent. were prostitutes.

A curious record has just been compiled by Professor Pellmann, of the University of Bonn. It relates to the career of a notorious drunkard, who was born in 1740 and died in 1800. In investigating her history her descendants were found to have numbered 834, of whom 709 have been traced from their youth. Of these seven were convicted of murder, seventy-six of other crimes, 142 were professional beggars, sixty-four lived on charity, and 181 women of the family led disreputable lives.

Perhaps nowhere is the doctrine of heredity more potent than in psychiatry, and in the statistics gathered and published by the

1. J. J. Berry : Medical Age.
2. Dugdale : The Jukes.—A study in crime.

different hospital systems, lunacy commissions and census reports one can gain some idea of the strength and solidity of this doctrine.

The New York State Lunacy Commission, with the large and well-conducted hospitals under its supervision, is able to furnish some figures on the frequency of heredity which must carry some weight and conviction even to those who are inclined to reject the doctrine or those who believe that its importance has been overestimated.

Studying carefully the reports of the eleven state hospitals for the year ending September 30, 1896, I found that the average percentage of heredity to be 25.38, while the percentage since 1888 is somewhat lower, being 23.56.[1]

In the census returns of 1890, of 70,340 insane, studied with reference to heredity, 22,077, or 31.38 per cent., had insane relatives; the number having insane fathers was 2,531, and insane mothers, 3,159; insane grandfathers, 784, insane grandmothers, 810; insane uncles, 2,408, insane aunts, 2,034; insane brothers, 3,630, insane sisters, 3,704; insane sons, 465, insane daughters, 480.[2]

An interesting medico-legal case that came to my attention in the Spring of 1891, where a young woman while in a state of psychical epilepsy, threw two children off a high trestle, killing one and seriously injuring another, disclosed a family tree quite remarkable for its neuropathic fruit. (See p. 8.) She was tried, adjudged irresponsible and sentenced to the Buffalo State Hospital. After a seclusion of four years she was released and immediately was married. The issue of this union can be surmised and the necessity for a more careful statute regulating marriage in New York state is obviously manifest.

It is a remarkable fact that notwithstanding the vital importance which attaches to the marriage contract, the rules of law which govern it are conflicting and contradictory and lack uniformity and harmony to a degree unknown in connection with any other species of contract. Every country, every state, prescribes the rules which shall prevail within its borders as to social order and domestic life. While some of the states have thrown some safeguards about the marriage ceremony, others like New York, for instance, have been most derelict in this particular and the highest court in this state

1. Percentage of heredity in New York State insane. Medicine, December, 1897.
2. Kellogg: Text-book of Mental Diseases (1897).

has decided, that a man and woman without going before a minister, or magistrate, without the presence of any person as a witness, with no previous public notice given, with no form of ceremony, civil or religious, and with no record or written evidence of the act, and merely by words of the present, may contract matrimony.[1]

The marriage laws of all the states are lax enough, but in New York, New Jersey, New Mexico, Montana, South Carolina and Wisconsin, even the marriage license, generally issued by the town clerk or registrar, is omitted. In the parish of Orleans, Louisiana, the board of health is designated to issue the license. At the last session of the Ohio legislature, Mr. Charles W. Parker introduced a measure which takes an advanced position on the subject and is the most progressive of any law yet proposed.

The bill provides that all persons seeking marriage license shall be examined by a board of three physicians, to be appointed by each county probate judge, who shall examine such persons to see that they are free from insanity, dipsomania, tuberculosis, cancer, venereal and other heredity diseases and are not criminals.

For this the author and even the state have been caustically criticised, but in reply to his critics Mr. Parker makes the following eloquent and timely reply: "It only aims to protect society and benefit the race. Dr. Doren has about 1,100 imbeciles under his charge as wards of the State. The majority of these unfortunates came into the world as the result of unwise marriages. Not a few of them are the direct result of persons of low intelligence marrying. It is a well-known fact that like begets like, drunkards begets drunkards and imbecile fathers have imbecile sons. A great deal is being said about degenerates and the rearing of professional criminals. In the majority of the cases their existence depends upon the marriage of persons who would not be permitted to contract such a sacred relation if some law similar to my bill were on the statute books."

The enactment of this bill into a law and reasonable enforcement may not act advantageously at first, since marriage laws change with geography and a ceremony in a neighboring state where no conditions are required would tend to nullify the objects sought after in the Ohio law.

With its hospitals for the insane and mentally infirm full to overflowing, its charity organisation societies complaining because of lack of funds on account of the increased demands upon their resources, its state prisons and reformatories crowded and a record

1. Snyder: Geography of Marriage.

of crimes committed during the past year unsurpassed, it is high time that the Empire State should follow quickly in the footsteps of its sister state and enact and then strictly enforce a law built on the same general plan as the Ohio law.

The other remedy proposed for grappling with the monster heredity, is asexualisation, which has of late received some attention by medical writers and at least by two state legislatures.

A bill has been introduced into the Michigan legislature by W. R. Edgar, providing for the castration of all inmates of the Michigan Home for the Feeble-Minded and Epileptic before their discharge; also for that of all persons convicted of a felony for the third time, and of those convicted of rape.

A similar bill has been introduced and favorably acted upon by committees of both houses of the Kansas legislature for the punishment of rape. Whether these measures have become laws, I have been unable to ascertain.

In 1881, Dr. C. H. Hughes, of St. Louis, first promulgated the castration plea in the following words: "For certain of the criminal class, and for certain defectives to whom liberty might be given, surgery suggests a plan less radical than that of Lycurgus, and more effective, and more appalling to real crime than guillotine or halter; equally conservative of mental and moral health, and in some instances absolutely curative of the individual."

It is difficult to say whether any practical headway will be made with this proposition, as it is radically different from any means of punishment now in vogue. It is a penalty unusual but not unreasonable, and deserves careful discussion and continued agitation.

Next in importance to the prevention of degeneracy is the redemption of degenerates and here *environment* is the all-important factor. Time, however, will not permit me to take up this question of the redemption or reclamation of the degenerate, but it can be carried out practically as readily as the methods proposed for prevention.

371 Delaware Avenue.

---

The Pain of Intestinal Obstruction.—A. H. Cordier (*Medical Herald*) says that a continuous and severe pain means a complete obstruction. A continuous mild pain with exacerbations implies a partial obstruction, such as chronic stricture or incomplete closing of acute intussusception. The location of the pain does not designate the site of the obstruction, unless of inflammatory origin.

## Figures and Tables

**Figure f1:**